# Attribution of physical complaints to the air disaster in Amsterdam by exposed rescue workers: an epidemiological study using historic cohorts

**DOI:** 10.1186/1471-2458-6-142

**Published:** 2006-05-30

**Authors:** Pauline Slottje, Nynke Smidt, Jos WR Twisk, Anja C Huizink, Anke B Witteveen, Willem van Mechelen, Tjabe Smid

**Affiliations:** 1 EMGO Institute, VU University Medical Center, Van der Boechorststraat 7, 1081 BT Amsterdam, The Netherlands; 2 Department of Public and Occupational Health, VU University Medical Center, Amsterdam, The Netherlands; 3 Department of Clinical Epidemiology and Biostatistics, Academic Medical Center, University of Amsterdam, The Netherlands; 4 Department of Clinical Epidemiology and Biostatistics, VU University Medical Center, Amsterdam, The Netherlands; 5 Department of Child and Adolescent Psychiatry, Erasmus Medical Center, Rotterdam, The Netherlands; 6 Department of Medical Psychology, VU University Medical Center, Amsterdam, The Netherlands; 7 KLM Health Services, Schiphol, The Netherlands

## Abstract

**Background:**

In 1992 a cargo aircraft crashed into a residential area of Amsterdam. A troublesome aftermath followed, with rumors on potential toxic exposures and health consequences. Health concerns remained even though no excess morbidity was predicted in retrospective risk evaluations. This study aimed to assess to what extent the rescue workers attribute long-term physical complaints to this disaster, including its aftermath, and to examine associations between such attribution and types of exposure and background variables.

**Methods:**

Historic cohort study that collected questionnaire data on occupational disaster exposure, attribution of physical complaints, and background variables on average 8.5 years post-disaster. For the present study the workers who were exposed to the disaster were selected from the historic cohort, i.e. the professional firefighters (n = 334), police officers (n = 834), and accident and wreckage investigators (n = 241) who performed disaster-related tasks.

**Results:**

Across the three occupational groups, a consistent percentage (ranging from 43% to 49%) of exposed workers with long-term physical complaints attributed these to the disaster, including its aftermath. Those with more physical complaints attributed these to a stronger degree. Multivariate logistic regression analyses showed that attribution was significantly more often reported by firefighters who rescued people, and by police officers who reported the identification and recovery of or search for victims and human remains, clean-up, or security and surveillance of the disaster area; who witnessed the immediate disaster scene; who had a close one affected by the disaster; and who perceived the disaster as the worst thing that ever happened to them. Age, sex and educational level were not significantly associated with attribution.

**Conclusion:**

This study provides further cross-sectional evidence for the role of causal attribution in post-disaster subjective physical health problems. After on average 8.5 years, almost a third (32%) of all the exposed workers, and almost half (45%) of the exposed workers with physical complaints, attributed these complaints to the disaster, including its aftermath. The similarity of the results across the occupational groups suggests a general rather than an occupation-specific attribution process. Longitudinal studies are needed to determine whether causal disaster attribution leads to persistence of post-disaster complaints and health care utilization.

## Background

In 1992 a cargo aircraft crashed into apartment buildings in a residential area of Amsterdam, killing 43 persons and destroying 266 apartments[1]. Through the years after the disaster, various alleged disaster-related exposures to hazardous materials and health consequences were reported in the media and publicly discussed [1-3]. No excess morbidity due to exposure to hazardous materials was predicted in retrospective risk evaluations [4,5], but health concerns remained among some of the residents and workers who were involved in the disaster [5,6]. Therefore, the epidemiological study air disaster in Amsterdam (ESADA) was initiated in 2000, to assess the relationship between occupational disaster exposure and the long-term health of rescue workers [7]. Previous results showed increased physical symptom rates among exposed workers compared to their colleagues who were not exposed to this disaster [8,9]. Although no clinical diagnostic data on these symptoms is available, extensive analysis of blood and urine samples did not reveal evidence for disaster-related pathological processes [8,9]. Subjective physical health problems without sufficient clinical or toxicological explanation are commonly referred to as "unexplained physical symptoms" in the literature. They are probably ubiquitous in the general population, but have also been reported after disasters and (perceived) noxious exposures in civilian [10-14] and military populations [15,16].

Causal attribution could play a role in the reported post-disaster subjective health problems. In general, people strive to understand the bodily sensations they detect and the causal attributions they adopt have been characterized as normalizing (i.e. benign sensations) as opposed to somatizing and psychologizing (i.e. symptoms) [17-19]. Further, a distinction is made between attributing symptoms to internal versus external (environmental) causes [20]. In times of stress and uncertainty about noxious exposures, people could be even more likely to attend to their bodies and detect bodily sensations (hypervigilance), and also to interpret the detected sensations as pathological (hypochondria) [21,22]. It has further been postulated that people affected by a disaster who perceive bodily sensations as pathological, are likely to attribute them to the disaster experience and related exposures, whether this is realistic or not [22-24]. According to Vasterman et al "there is a strong relationship between the symptomatology seen in the aftermath of disasters and medically unexplained physical symptoms in the general population. The only difference is that, after a disaster, the symptoms are attributed to the event" [3].

In addition to its potential role in post-disaster subjective health problems, causal attribution may also affect the course of physical symptoms. Strong attributions to somatic causes and environmental exposures, for example, have been shown to be associated with a worse prognosis in patients with chronic fatigue syndrome and in other patients with medically unexplained physical symptoms [25-30]. The medical expenses of these patients with sustained physical symptoms could be higher, because of their frequent visits to various medical specialists, who usually cannot confirm the patient's conviction [31,32].

Thus, causal attributions could affect physical health perception, functioning, and health care utilization, both in general and after disasters. However, little is known about the causal attributions of those involved in disasters. The main aim of the present study is to assess the extent to which the rescue workers, who were occupationally exposed to the air disaster in Amsterdam, attribute their long-term physical complaints to this disaster, including its aftermath. Because those who attribute physical complaints to a disaster may need a different approach in after care programs, an attempt is also made to characterize those with such attribution. To this end, the present study also examines which factors are associated with attribution, in terms of types of exposure and background characteristics. These characteristics might help in targeting a tailored after care program to those who attribute physical complaints to the disaster.

## Methods

### Participants and data collection

The present study is part of the Epidemiological Study Air Disaster in Amsterdam (ESADA), of which the study design has been published previously [7]. The ESADA is a historic cohort study, using self-reported exposure status. The study population consists of a historic cohort of rescue workers, including both the workers who were occupationally exposed to this disaster (i.e. who reported one or more disaster-related tasks), and their colleagues who were not exposed to it. The overall participation rate was 70% of those traced and invited to join in the study (n = 3742). For the purpose of the present study the participating 1409 exposed workers were selected. This concerns three occupational groups: (1) professional firefighters (n = 334) working at the Amsterdam fire department at the time of the disaster; (2) police officers (n = 834) working at the Amsterdam-Amstelland regional police force at the time of the disaster and at the start of the study; and (3) accident and wreckage investigators (so-called hangar workers) (n = 241), who worked for one of the departments involved in the transport, security and sorting of the wreckage at Schiphol Airport at the time of the disaster.

The medical ethics committees of the medical centers involved in the ESADA approved the study protocol (i.e. the VU University Medical Center and the Onze Lieve Vrouwe Gasthuis in Amsterdam). Participants signed informed consent and participated voluntarily. Data were collected from January 2000 to March 2002, i.e. on average 8.5 years post-disaster. All the data used in the present study were assessed by means of questionnaires and were entered twice, after which inconsistencies were reviewed and any mistakes rectified.

### Long-term physical complaints and attribution thereof

Workers were asked to indicate whether they currently had physical complaints on a four-point scale (very many, many, few, or none). Those who reported having physical complaints were subsequently asked to indicate to what extent they thought these were related to the air disaster, including its aftermath, on a four-point scale (a very strong, strong, weak, or no relationship). Attribution was defined as reporting a weak, strong or very strong relationship. Workers who attributed physical complaints, were asked to specify these complaints, i.e. skin, back, joints, shortness of breath or lung problems, fatigue, headaches, or other.

In addition, all workers were asked to indicate the current absence or presence of 34 physical symptoms (from a questionnaire that was constructed specifically for this study [33]), and to complete the 20-item Checklist Individual Strength [34] on fatigue-related symptoms, the presence of which was defined as a total score above 76 [35].

### Type of exposure to the disaster

Type of exposure is characterized according to the following variables:

a. Disaster-related tasks: having performed the following tasks: rescuing people; identification and recovery of or search for victims and human remains; firefighting; clean-up of the disaster site; security and surveillance of the disaster area; supporting injured victims and workers; and sorting of the wreckage in a hangar at Schiphol Airport.

b. Witnessed the immediate disaster scene: having seen the disaster scene within the first hours after the crash, or when the wreckage was still there.

c. Having a close one affected by the disaster: having a close or beloved one (i.e. family members, relatives, friends or acquaintances) who was affected by the disaster (i.e. in life-threatening danger; injured; destroyed apartment; died; or affected in any other way).

d. Perceived severity of the disaster: workers were asked to complete the following statement with one of the offered answers: "The air disaster and its aftermath was...": (i) not bad; (ii) quite bad; (iii) terrible, but not the worst thing that ever happened to me (abbreviated to "terrible" from now on); or (iv) the worst thing that ever happened to me (abbreviated to "worst thing ever" from now on).

### Background characteristics

Background characteristics were categorized as follows: age at time of assessment (young versus old [≥ median age of exposed workers with physical complaints per occupational group]); sex (male versus female); and highest level of completed education (low [no education, elementary school, lower vocational education, or lower general secondary education], intermediate [intermediate vocational education, higher general secondary education, pre-university education], versus high [higher vocational education, university]). Data on age and sex were complete. For level of education an additional missing category was used in the statistical analysis, to prevent excluding these workers (6%).

### Statistical analysis

The following statistical analyses were performed among the workers with physical health complaints, using SPSS (version 10.1) and considering two-sided P-values less than 0.05 as statistically significant. Associations between the degree of physical complaints ([very] many versus few) and attribution thereof (a [very] strong, or weak versus no relationship) were analyzed by means of Pearson χ^2 ^tests. Logistic regression was used to analyze associations between the specific types of physical symptoms (dependent variable: present versus absent) and the extent of attribution (categorical independent variable: [very] strong, weak versus no relationship).

Logistic regression was also used to analyze associations between attribution (dichotomized into yes [very strong, strong or weak relationship] and no [no relationship]) and the following independent variables: the applicable disaster-related tasks, having witnessed the immediate disaster scene, having a close one affected by the disaster (each coded as yes versus no), perceived severity of disaster experience (categorical with "worst thing ever" as reference category), and the background characteristics. In this logistic regression analysis, the independent variables were first introduced separately in "univariate" models, after which they were all introduced together and those with P > 0.10 were subsequently removed in a step-wise backward manner, until only those with P ≤ 0.10 were retained in the final "multivariate" model.

## Results

### Background characteristics of exposed workers

The firefighters, all male, had a mean age of 51.4 years (SD 5.9), and 59%, 28%, and 6% of them reported a low, intermediate, and high level of education, respectively (8% no data on education). The mean age of the police officers was 44.0 years (SD 6.2); 89% of them were male, and 21%, 53%, and 21% of them reported a low, intermediate, and high level of education, respectively (6% no data on education). The hangar workers, all male, had a mean age of 43.9 years (SD 7.8), and 43%, 44%, and 8% of them reported a low, intermediate, and high level of education, respectively (5% no data on education).

### Prevalence of long-term physical complaints and attribution thereof

The prevalence of long-term physical complaints and attribution thereof was very similar across the three occupational groups (Table 1). 72% of all exposed workers reported long-term physical complaints, of whom the majority reported to have few physical complaints. 45% of the workers with long-term physical complaints attributed these to the disaster to some extent. 23% of the workers who attributed physical symptoms to the disaster reported this to be a (very) strong relationship. Workers with more physical complaints were more likely to attribute these complains to a stronger degree to the disaster (P < 0.0001 within each occupational group).

The top three of types of physical complaints most frequently attributed to the disaster by firefighters, police officers and hangar workers were: skin complaints (58, 50% and 50%, respectively), fatigue (42%, 47% and 70%, respectively), and joint complaints (47%, 35% and 54%, respectively). The prevalence rates of these three types and the other types of physical symptoms were also positively associated with the extent of attribution of physical complaints to the disaster (Table 2).

### Factors associated with attribution of physical complaints

In the univariate analysis of firefighters with physical complaints, attribution was significantly associated with rescuing people, firefighting, supporting injured victims and workers, and having witnessed the immediate disaster site (Table 3). However, only rescuing people remained significant in the multivariate analysis, while supporting injured victims and workers, and having witnessed the immediate disaster scene had P ≤ 0.10. The effect sizes of these types of exposure were similar, but tended to be somewhat lower in multivariate compared to univariate analyses. Background characteristics were not significantly associated with attribution.

With respect to the police officers, attribution was significantly associated with all the types of exposure both in univariate and in multivariate analyses, except for identification and recovery of or search for victims and human remains in univariate analysis, and rescuing and supporting people in multivariate analysis (Table 4). Some multivariate odds ratio's were somewhat higher, while others were somewhat lower compared to the univariate ones. Background characteristics were not significantly associated with attribution among police officers.

Regarding the hangar workers, none of the types of exposure or background characteristics were significantly associated with attribution, although the analysis of education level indicated that hangar workers with intermediate and low levels of education were less likely to attribute physical complaints to the disaster than those with a high level of education (P = 0.05) (Table 5).

## Discussion

The aim of the present study was primarily to assess the extent to which exposed workers attributed their long-term physical health complaints to the disaster, including its aftermath, and, secondary, to characterize those who did report such attribution. The results were remarkably similar across the three occupational groups despite their distinct occupational involvement in the disaster. The similarity of results concerned (a) the prevalence of long-term physical health complaints (varying from 70 to 79%, depending on the occupational group); (b) the proportion attributing these complaints to the disaster, including its aftermath, to a weak (32–38%), strong (7–9%), or very strong (1–2%) degree; (c) the types of physical complaints they attributed (the top three being skin complaints, fatigue, and joint pain in each group); (d) the positive associations between the extent of attribution and the severity of physical complaints as well as the prevalence of various physical symptoms, including the abovementioned top three of complaints.

A remaining intriguing question is to what specific aspects of the disaster and its aftermath the workers attributed their physical complaints, and to what extent these attributions are realistic. The finding that the majority of workers who attributed physical complaints to the disaster reported this to be a weak relationship might indicate that these workers could simply not exclude the possibility of such a relationship, rather than that they had explicit causal ideas about it. Moreover, the similarity of the results across the occupational groups could indicate that attributing physical complaints to the disaster depended on general factors rather than on occupation-specific factors.

There are three ways through which disasters may result in long-term health problems: direct physical harm (such as burns, injuries), exposure to psychotraumatic and otherwise stressful events, and exposure to hazardous materials. Long-term health consequences of acute physical harm seems unlikely in this case, because only 0.4% of the exposed police officers and none of the exposed firefighters and hangar workers reported to have been personally injured.

Regarding psychotraumatic events, the exposure types "rescuing people" and "identification and recovery of or search for victims and human remains" have previously been identified as potentially psychotraumatic by experts on posttraumatic stress disorder [7]. These tasks were statistically significantly associated with attribution among firefighters and police officers, respectively. Post-hoc analyses nevertheless showed that the long-term prevalence of high levels of posttraumatic stress symptoms was only about 6% among firefighters and police officers and that inclusion of these symptoms in the multivariate models did not essentially change the associations between attribution and types of exposure (data not shown). Thus, if any, psychotrauma probably only plays a minor role in the attribution process in this case.

With respect to exposure to hazardous materials, retrospective risk evaluations predicted no excess in chronic morbidity due to noxious exposures related to air disaster [5,6]. Moreover, previous comparisons of these exposed workers and their colleagues who were not exposed to the air disaster revealed no evidence for disaster-related pathological processes based on extensive clinical analysis of blood and urine samples [8,9]. The comparison of exposed and nonexposed workers did show that exposed workers reported various physical symptoms statistically significantly more often [8,9]. These previously reported results therefore resemble a phenomenon commonly referred to as "unexplained physical symptoms". Such phenomena have also been demonstrated in civilian and military populations after disastrous events and after perceived exposure to hazardous materials [10-16]. The absence of epidemiological evidence for disaster-related pathological processes that could explain the excess physical symptoms among exposed workers, however, does not imply that the worker's appraisal of a relationship between their physical complaints and the disaster is unjust or fictitious.

One likely candidate for a general factor affecting health perception and attribution is the complex aftermath of this disaster. The aftermath was characterized by numerous media reports and public discussions on alleged exposure to hazardous materials and health consequences [1-3]. A variety of hazardous exposures sequentially emerged in the public debate and were coined as explanation for any type of post-disaster health problem. The rumors were most probably also discussed among the exposed rescue workers and they could have affected health perception and attribution of health complaints among exposed workers in two ways. Firstly, they might have contributed to perceiving the disaster as a health threat. Previous studies have argued that considering an environmental factor as harmful to health is important for subjective health. For example, in a study after the Chernobyl disaster, risk perception was suggested to play a mediating role between (perceived) exposure and subjective health problems [36]. Also, in comparisons of residents of potentially contaminated and control areas, residents who consider the contamination as harmful to health are the once that most often report lower levels of subjective health [37,38].

Secondly, the rumors and speculations might have contributed to sustained uncertainty. In an attempt to reduce such uncertainty, workers may have been more inclined to refer to the actions of "similar others", i.e. symptomatic colleagues who attributed health problems to the disaster, to decide how to act themselves [14]. Vastermans et al previously suggested that "there is a strong relationship between the symptomatology seen in the aftermath of disasters and medically unexplained physical symptoms in the general population. The only difference is that, after a disaster, the symptoms are attributed to the event" [3]. As more people adopt that attribution, it might become socially accepted and spread across the affected population. Such spreading of attribution might be enhanced in cases of distrust in official information on noxious exposures, as was the case in the air disaster for some of the affected people. An interesting remaining question is whether awareness of the attributions of others could also lead to spreading of particular symptoms, i.e. that more people perceive the same symptoms if it becomes generally known that similar others have attributed those particular symptoms to the disaster. In this respect, a parallel can also be drawn with so-called Mass Psychogenic/Sociogenic Illness, in which a short-lived spreading of particular physical symptoms in communities has been postulated [39]. However, no evidence was found for spreading of particular physical symptoms, because the prevalence rates of a wide variety of physical symptoms was higher among exposed compared to nonexposed workers, and because the prevalence rates of various physical symptoms were also positively associated with the extent of attribution to the air disaster in Amsterdam.

Besides genuinely perceiving a relationship between physical health complaints and the disaster, some exposed workers might alternatively have been motivated to report such a relationship for reasons of secondary gain, such as recognition, attention, and financial compensation. Unfortunately, no data are available to further look into this matter.

In an attempt to characterize those who attributed physical complaints to the disaster, associations with types of exposure and background characteristics were examined. As discussed above, the similarity of the results across the different occupational groups suggest that general rather than occupation-specific factors contributed to attributing physical complaints to the disaster. Nevertheless, the multivariate logistic regression analyses showed that attribution was significantly associated with some types of exposure within a particular occupational group. This concerned rescuing people among firefighters and almost all types of exposure among police officers, i.e. three of the five tasks, having witnessed the immediate disaster scene, having a close one affected by the disaster, and perceiving the disaster and its aftermath as the worst thing that ever happened to them. Because most of the firefighters reported multiple tasks, whereas most of the police officers reported one of the specified tasks, it may have been easier to detect independent effects of particular tasks among police officers. The difference in sample size between the groups may also have contributed to this. No significant associations between attribution and types of exposure were found among hangar workers, but the analysis was limited to three variables: sorting the wreckage, witnessing the immediate disaster scene, and having a close one affected by the disaster. Only a few hangar workers reported the latter two items.

A positive association was also found between attribution and the perceived severity of the experience of the disaster, including its aftermath, which was significantly only in the largest of the studied groups; the police officers. It was decided to regard the perceived severity of the disaster primarily as an exposure variable, which putatively encompassed a general appraisal of the experience of their involvement in the disaster and its aftermath. It might alternatively have been regarded as an outcome variable, which would imply that the perceived severity depended on workers' appraisal of the health impact of this disaster. Both these interpretations could partly explain the positive associations seen between attribution and the severity of the disaster experience.

The analyses also revealed that attribution was not significantly associated with the background characteristics age, level of education, and sex. Sex could only be taken in consideration for police officers, because all the included firefighters and hangar workers were male. The results regarding age and sex are not in line with those of Stuart et al. (2003) who found that among veterans of the first Gulf War, females and those who were older (age 32 to 61 years) were more likely to report belief in exposure to terrorist agents (nerve or mustard gas) [40]. In that study, belief in exposure to terrorist agents was also associated with degree of exposure, i.e. reporting more exposures (non-nerve or mustard gas) to potentially toxic agents and traumatic events.

Donker et al. (2002) previously reported on a self-selected group (n = 553) of residents, rescue workers and others affected by the air disaster in Amsterdam [6]. On their own initiative, these individuals called a toll free call centre (in June-July 1998) to report the health complaints they attributed to this disaster. Of the three physical complaints that were most frequently attributed to the disaster in the present study, fatigue and dry skin were also in the top ten of spontaneously reported health complaints at the call centre (reported by 45% and 13% of the callers, respectively). For 3% and 15% of these two symptoms, respectively, a relationship between the disaster and these particular symptoms was considered to be realistic according to the general practitioners of the callers with these complaints.

In the present study, no clinical judgment of the perceived relationship between physical complaints and the air disaster is available. However, irrespective of the credibility from a clinical perspective, the causal attributions of exposed workers could affect the prognosis of post-disaster health complaints [25-30], functioning, and the utilization of health care [31,32]. For example, the prevalence of role-limitations due to physical problems was positively and significantly associated with the strength of attribution to the disaster in each of the occupational groups (data not shown). It could thus be relevant to establish whether people involved in disasters (or other events with perceived exposure) attribute any health complaints to that disaster, because they may need a different approach in after care programs. Due to the cross-sectional nature of the present study, it was unfortunately not possible to assess the longitudinal course of health complaints, attribution thereof, and the health care used for them.

The strength of the present study is that it is based on a historically-defined study population consisting of all the rescue workers who were occupationally exposed to the disaster, irrespective of their health status. Therefore it tentatively provides a representative estimate of the prevalence of long-term physical complaints (72%) and attribution thereof among all the exposed professional firefighters, police officers and hangar workers. In total, 32% of all the exposed workers, and 45% of the exposed workers with physical complaints, attributed their complaints to the disaster, including its aftermath.

Some limitations should also be mentioned. First of all, as in all cross-sectional studies, the study design precludes drawing inferences on the direction or causality of the associations, for example regarding the positive associations between attribution and the severity of physical health complaints as well as the perceived severity of the disaster experience.

Secondly, recall or reporting bias may have biased the associations between the self-reported types of exposure and attribution. Furthermore, the types of exposure are likely to be partly inter-related, e.g. rescuers probably also witnessed the immediate disaster scene. Therefore, in addition to univariate associations with attribution, the types of exposure were entered in a multivariate model from which those with P > 0.10 were eliminated in a step-wise, backward manner. The associations that were found univariately remained essentially the same in the multivariate analysis, thus indicating independent associations between these types of exposure and attribution.

A final point of attention is that, although almost all data were virtually complete, the data on the item "which type of physical complaints" the workers attributed to the disaster was only available for 18%, 99.6% and 53% of the firefighters, police officers and hangar workers, respectively, who attributed any physical complaints to the disaster and its aftermath. This was due to administrative difficulties at the start of the data collection. While the data on this item might be biased by its incompleteness, there is no reason to believe that the workers who were assessed in the first part would have attributed a different type of physical symptom to the disaster than the workers who were assessed in the last part of the data collection period. In addition, the top three of most frequently attributed physical complaints was the same while the completion rate varied considerably across the three occupational groups.

## Conclusion

In conclusion, this study provides further cross-sectional evidence for the role of causal attribution in post-disaster subjective physical health problems, which lack sufficient clinical or toxicological explanation. After on average 8.5 years, almost a third (32%) of all the exposed workers, and almost half (45%) of the exposed workers with physical complaints, attributed physical complaints to the disaster, including its aftermath. The similarity of the results across the occupational groups suggests that this attribution process was a general rather than an occupation-specific phenomenon, tentatively fed by the rumors on noxious exposures and health consequences in the aftermath of the disaster. Longitudinal studies are needed to determine whether attribution of post-disaster health complaints leads to persistence of health complaints and health care utilization.

## Competing interests

The author(s) declare that they have no competing interests.

## Authors' contributions

All authors participated in the multidisciplinary ESADA project team of the EMGO Institute, provided comments on the draft versions and approved the final manuscript. In addition, PS drafted the manuscript and performed the statistical analyses; NS supervised the second part of the ESADA; JT contributed to the design and supervised the statistical analyses; AH coordinated the acquisition of data and supervised the first part of the ESADA; AW contributed to the statistical analysis of posttraumatic stress symptoms and the Checklist Individual Strength; WM contributed to the design and is Vice-President of the ESADA project team; and TS conceived of the study, and participated in its design and coordination as President of the ESADA project team.

## Pre-publication history

The pre-publication history for this paper can be accessed here:



## Figures and Tables

**Table 1:** Prevalence of physical complaints and attribution thereof by exposed rescue workers

	**Firefighters (n = 334)**	**Police officers (n = 834)**	**Hangar workers (n = 241)**
**Physical complaints:**†			
Yes (few through very many)	73 %	70 %	79 %
Few physical complaints	61 %	58 %	62 %
Many physical complaints	12 %	11 %	14 %
Very many physical complaints	0.6 %	1 %	2 %
**Attribution to disaster and aftermath:**‡			
Yes (weak through very strong)	46 %	43 %	49 %
Weak relationship	38 %	32 %	38 %
Strong relationship	7 %	9 %	8 %
Very strong relationship	0.9 %	2 %	2 %

†Percentage of all the exposed workers per occupational group; missing data on physical complaints for 4 exposed police officers and 4 exposed hangar workers.

‡Percentage of workers with physical complaints per occupational group; missing data on attribution for 11, 35, and 8 of the exposed firefighters, police officers and hangar workers with physical complaints, respectively.

**Table 2:** Prevalence of physical complaints according to the extent of attribution

	Attribution:†	Skin:‡	Fatigue:§	Joint:#	Back¶	Respiratory complaints ||	Headache^
Firefighters with physical complaints (n = 243)	No (n = 125)	52 %	7.2 %	44 %	30 %	29 %	32 %
	Weak (n = 89)	65 %	23 %*	61 %*	42 %	44 %*	45 %*
	(Very) strong (n = 18)	89 %*	44 %*	78 %*	33 %	56 %*	33 %
Police officers with physical complaints (n = 580)	No (n = 310)	47 %	14 %*	34 %	27 %	29 %	36 %
	A little (n = 175)	74 %*	31 %*	39 %	29 %	45 %*	53 %*
	(Very) strong (n = 60)	82 %*	42 %*	47 %	37 %	52 %*	62 %*
Hangar workers with physical complaints (n = 178)	No (n = 91)	58 %	14 %	37 %	36 %	34 %	40 %
	A little (n = 68)	72 %	29 %*	62 %*	31 %	50 %*	49 %
	(Very) strong (n = 19)	74 %	63 %*	68 %*	58 %	47 %	47 %

†Attribution: No, a weak, and a (very) strong relationship between physical complaints and the air disaster in Amsterdam, including its aftermath, respectively.

‡Skin: any of 13 listed skin complaints (such as eczema, skin infections, or "other skin complaints"): yes versus no.

§Fatigue: Checklist Individual Strength, total score >76: yes versus no.

#Joint: pain in any joints (except low back pain), for at least three consecutive months: yes versus no.

¶Back: low back pain for at least three consecutive months: yes versus no.

||One of six respiratory symptoms (such as shortness of breath and chronic cough): yes versus no.

^Having been distressed by headaches in the past 7 days: a little through extremely versus not at all.

*p < 0.05 compared to no attribution, using logistic regression (complaint yes versus no).

**Table 3:** Associations between attribution and types of exposure and background characteristics in exposed firefighters with long-term physical complaints

	Prevalence (column %)		Odds ratio (95% confidence interval)	
	
	Attribution† (n = 107)	No attribution† (n = 125)	Univariate analysis‡	Multivariate analysis§
**Type of exposure:**				
Rescuing people	62	40	2.5 (1.5–4.2)**	2.0 (1.2–3.5)*
Identification and recovery of or search for victims and human remains	13	18	0.69 (0.33–1.4)	-
Firefighting	71	54	2.1 (1.2–3.6)§	-
Clean-up of disaster site	55	59	0.83 (0.49–1.4)	-
Security and surveillance of disaster area	5	2	3.0 (0.57–15.8)	-
Supporting injured victims and workers	16	7	2.7 (1.1–6.6)*	2.4 (0.95–5.9)
Witnessed immediate disaster scene	82	67	2.3 (1.2–4.2)*	1.9 (0.97–3.6)
Close one affected by disaster	3	6	0.49 (0.12–1.9)	-
Perceived severity disaster:				
- not bad	7	12	0.38 (0.12–1.2)	-
- quite bad	11	10	0.75 (0.26–2.2)	-
- terrible	67	67	0.70 (0.31–1.5)	-
- worst thing ever	15	10	Reference	-
**Background characteristics:**				
Age (young)	47	53	0.78 (0.47–1.3)	-
Education				
- high	10	5	Reference	-
- intermediate	26	26	0.46 (0.15–1.4)	-
- low	58	60	0.45 (0.16–1.3)	-

†Attribution (a weak through very strong relationship) versus no attribution (no relationship between physical complaints and the air disaster in Amsterdam and its aftermath). ‡Number of firefighters included in the univariate analyses ranged from 226 to 232 due to occasional missing values. §The final multivariate model was based on 228 firefighters and includes only those independent variables with P ≤ 0.10. *P < 0.05; ** P < 0.01.

**Table 4:** Associations between attribution and types of exposure and background characteristics in exposed police officers with long-term physical complaints

	Prevalence (column %)		Odds ratio (95% confidence interval)	
	
	Attribution†(n = 235)	No attribution† (n = 310)	Univariate analysis‡	Multivariate analysis§
**Type of exposure:**				
Rescuing people	21	10	2.4 (1.5–3.9)***	1.6 (0.96–2.7)
Identification and recovery of or search for victims and human remains	12	8	1.7 (0.95–3.0)	2.2 (1.1–4.3)**
Clean-up of disaster site	7	3	2.8 (1.2–6.6)*	2.8 (1.1–7.0)*
Security and surveillance of disaster area	83	75	1.7 (1.1–2.5)*	1.8 (1.1–3.0)*
Supporting injured victims and workers	31	22	1.6 (1.1–2.4)*	-
Witnessed immediate disaster scene	83	70	2.1 (1.4–3.2)***	1.8 (1.1–3.0)*
Close one affected by disaster	11	4	2.8 (1.4–5.7)**	3.1 (1.5–6.5)**
Perceived severity disaster				
- not bad	3	10	0.20 (0.08–0.48)***	0.23 (0.09–0.57)**
- quite bad	9	10	0.57 (0.29–1.1)	0.64 (0.32–1.3)
- terrible	62	61	0.70 (0.46–1.1)	0.73 (0.47–1.1)
- worst thing ever	26	18	Reference	Reference
**Background characteristics:**				
Age (young)	50	52	0.93 (0.66–1.3)	-
Education				
- high	21	21	Reference	-
- intermediate	49	56	0.87 (0.56–1.3)	-
- low	22	18	1.2 (0.70–2.0)	-
Sex (male)	91	85	1.9 (1.0–3.2)*	-

†Attribution (little through a very strong relationship) versus no attribution (no relationship between physical complaints and the air disaster in Amsterdam and its aftermath). ‡Number of police officers included in the univariate analyses ranged from 536 to 545 due to occasional missing values. §The final multivariate model was based on 529 police officers and includes only those independent variables with P ≤ 0.10. *P < 0.05; ** P < 0.01; *** P < 0.001.

**Table 5:** Associations between attribution and types of exposure and background characteristics in exposed hangar workers with long-term physical complaints

	Prevalence (column %)		Odds ratio (95% confidence interval)	
	
	Attribution† (n = 87)	No attribution† (n = 91)	Univariate analysis‡	Multivariate analysis§
**Type of exposure:**				
Sort the wreckage in hangar	62	70	0.69 (0.37–1.3)	-
Witnessed immediate disaster scene	8	11	0.71 (0.26–2.0)	-
Close one affected by the disaster	5	7	0.68 (0.19–2.5)	-
Perceived severity disaster				
- not bad	9	16	0.35 (0.12–1.1)	-
- quite bad	15	18	0.50 (0.18–1.4)	-
- terrible	51	52	0.58 (0.26–1.3)	-
- worst thing ever	25	15	Reference	-
**Background characteristics:**				
Age (young)	44	58	0.56 (0.31–1.0)	-
Education				
- high	9	6	Reference	Reference
- intermediate	32	53	0.37 (0.11–1.2)	0.37 (0.11–1.2)
- low	53	37	0.85 (0.25–2.8)	0.85 (0.25–2.8)

†Attribution (little through a very strong relationship) versus no attribution (no relationship between physical complaints and the air disaster in Amsterdam and its aftermath). ‡Number of hangar workers included in the univariate analyses ranged from 174 to 178 due to occasional missing values. §The final multivariate model was based on 169 hangar workers and includes only those independent variables with P ≤ 0.10.
